# 1-Chloro-2-methyl-3-nitro­benzene

**DOI:** 10.1107/S1600536811004466

**Published:** 2011-02-12

**Authors:** Matthew A. Pearce, Joseph M. Tanski

**Affiliations:** a Department of Chemistry, Vassar College, Poughkeepsie, NY 12604, USA

## Abstract

In the title compound, C_7_H_6_ClNO_2_, the chloro, methyl and nitro substituents are situated next to each other in this order on the benzene ring, with the mean plane of the nitro group twisted away from the mean plane of the benzene ring by 38.81 (5)°.

## Related literature

For information on industrial chemicals, see: Chloro­nitro­toluenes (2010 ▶). For the use of the title compound as a starting material in the synthesis of 7-chlorovasicine (pyrrolo[2,1-*b*]quinazolin-3-ol, 8-chloro-1,2,3,9-tetrahydro), see: Southwick & Cremer (1959 ▶). For the toxic effects of the title compound on *D. magna*, see: Ramos *et al.* (2001 ▶) and on *T. pyriformis*, see: Schultz (1999 ▶); Katritzky *et al.* (2003 ▶). For a related structure, see: Liu & Du (2008 ▶).
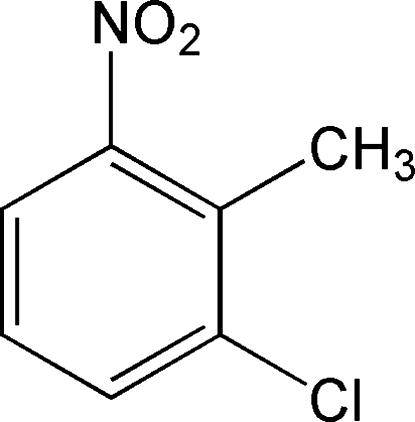


## Experimental

### 

#### Crystal data

C_7_H_6_ClNO_2_
*M* *_r_* = 171.58
Orthorhombic, 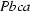

*a* = 7.3061 (5) Å
*b* = 13.8392 (9) Å
*c* = 14.6799 (10) Å
*V* = 1484.29 (17) Å^3^
*Z* = 8
Mo *K*α radiation
μ = 0.46 mm^−1^
*T* = 125 K
0.32 × 0.20 × 0.10 mm

#### Data collection

Bruker APEXII CCD diffractometer
Absorption correction: multi-scan (*SADABS*; Bruker, 2007 ▶) *T*_min_ = 0.868, *T*_max_ = 0.956
22310 measured reflections
2271 independent reflections
1963 reflections with *I* > 2σ(*I*)
*R*_int_ = 0.038

#### Refinement

*R*[*F* ^2^ > 2σ(*F* ^2^)] = 0.031
*wR*(*F* ^2^) = 0.090
*S* = 1.05
2271 reflections
101 parameters
H-atom parameters constrained
Δρ_max_ = 0.35 e Å^−3^
Δρ_min_ = −0.38 e Å^−3^

### 

Data collection: *APEX2* (Bruker, 2007 ▶); cell refinement: *SAINT* (Bruker, 2007 ▶); data reduction: *SAINT*; program(s) used to solve structure: *SHELXS97* (Sheldrick, 2008 ▶); program(s) used to refine structure: *SHELXL97* (Sheldrick, 2008 ▶); molecular graphics: *SHELXTL* (Sheldrick, 2008 ▶); software used to prepare material for publication: *SHELXTL*.

## Supplementary Material

Crystal structure: contains datablocks I, global. DOI: 10.1107/S1600536811004466/jj2075sup1.cif

Structure factors: contains datablocks I. DOI: 10.1107/S1600536811004466/jj2075Isup2.hkl

Additional supplementary materials:  crystallographic information; 3D view; checkCIF report

## Figures and Tables

**Fig. 1.**
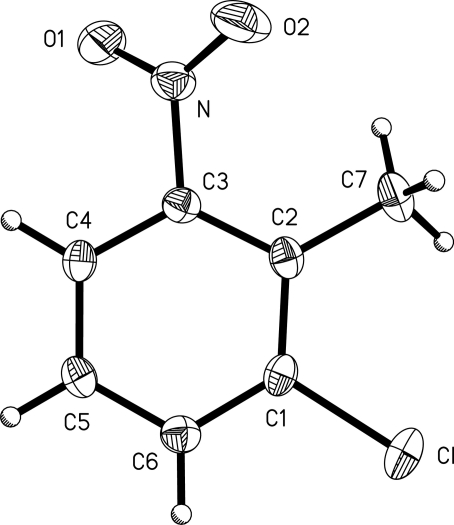
A view of the title compound, with displacement ellipsoids shown at the 50% probability level.

## supplementary crystallographic information

### Comment

The title compound, C_7_H_6_ClNO_2_, also known as 2-chloro-6-nitrotoluene, is a common intermediate in the synthesis of industrial chemicals (Chloronitrotoluenes, 2010), as well as in pharmaceuticals such as the bronchodilatory compound vasicine (Southwick *et al.*, 1959). 1-chloro-2-methyl-3-nitrobenzene is relatively toxic to biological species such as the freashwater flea *D. magna* and freshwater protozoa *T. pyriformis*, which suggests that this compound could become a harmful pollutant (Katritzky *et al.*, 2003; Schultz, 1999; Ramos *et al.*, 2001; Chloronitrotoluenes, 2010).

C_7_H_6_ClNO_2_, (I), contains an aromatic ring with chloro, methyl and nitro substituents arranged in this order next to one another, The C—Cl and C—N bond lengths and angles in (I) are very close to those found in a similar structure (Liu & Du, 2008).  The central methyl group interacts sterically with the neighboring chloro and nitro groups, as evidenced by the N—C3—C4 and Cl—C1—C6 angles of 115.0 (1)° and 116.78 (8)°, respectively.  These angles are compressed from the ideal *sp*^2^ - hybridized carbon atom.  The mean plane of the nitro group is twisted away from the mean plane of the aromatic ring by 38.81 (5)°.

### Experimental

Crystalline1-chloro-2-methyl-3-nitrobenzene was purchase from Aldrich Chemical Company, USA.

### Refinement

All non-hydrogen atoms were refined anisotropically. Hydrogen atoms on carbon were included in calculated positions and refined using a riding model at C–H = 0.95 or 0.98 Å and *U*_iso_(H) = 1.2 or 1.5 × *U*_eq_(C) of the aryl and methyl C-atoms, respectively. The extinction parameter (EXTI) refined to zero and was removed from the refinement.

### Crystal data

**Table:**

C_7_H_6_ClNO_2_	*F*(000) = 704
*M**_r_* = 171.58	*D*_x_ = 1.536 Mg m^−^^3^
Orthorhombic, *P**b**c**a*	Mo *K*α radiation, λ = 0.71073 Å
Hall symbol: -P 2ac 2ab	Cell parameters from 9979 reflections
*a* = 7.3061 (5) Å	θ = 2.8–30.5°
*b* = 13.8392 (9) Å	µ = 0.46 mm^−^^1^
*c* = 14.6799 (10) Å	*T* = 125 K
*V* = 1484.29 (17) Å^3^	Block, colorless
*Z* = 8	0.32 × 0.20 × 0.10 mm

### Data collection

**Table:**

Bruker APEXII CCD diffractometer	2271 independent reflections
Radiation source: fine-focus sealed tube	1963 reflections with *I* > 2σ(*I*)
graphite	*R*_int_ = 0.038
φ and ω scans	θ_max_ = 30.5°, θ_min_ = 2.8°
Absorption correction: multi-scan (*SADABS*; Bruker, 2007)	*h* = −10→10
*T*_min_ = 0.868, *T*_max_ = 0.956	*k* = −19→19
22310 measured reflections	*l* = −20→20

### Refinement

**Table:**

Refinement on *F*^2^	Primary atom site location: structure-invariant direct methods
Least-squares matrix: full	Secondary atom site location: difference Fourier map
*R*[*F*^2^ > 2σ(*F*^2^)] = 0.031	Hydrogen site location: inferred from neighbouring sites
*wR*(*F*^2^) = 0.090	H-atom parameters constrained
*S* = 1.05	*w* = 1/[σ^2^(*F*_o_^2^) + (0.0501*P*)^2^ + 0.4184*P*] where *P* = (*F*_o_^2^ + 2*F*_c_^2^)/3
2271 reflections	(Δ/σ)_max_ = 0.001
101 parameters	Δρ_max_ = 0.35 e Å^−^^3^
0 restraints	Δρ_min_ = −0.38 e Å^−^^3^

### Special details

**Table:**

Geometry. All e.s.d.'s (except the e.s.d. in the dihedral angle between two l.s. planes) are estimated using the full covariance matrix. The cell e.s.d.'s are taken into account individually in the estimation of e.s.d.'s in distances, angles and torsion angles; correlations between e.s.d.'s in cell parameters are only used when they are defined by crystal symmetry. An approximate (isotropic) treatment of cell e.s.d.'s is used for estimating e.s.d.'s involving l.s. planes.
Refinement. Refinement of *F*^2^ against ALL reflections. The weighted *R*-factor *wR* and goodness of fit *S* are based on *F*^2^, conventional *R*-factors *R* are based on *F*, with *F* set to zero for negative *F*^2^. The threshold expression of *F*^2^ > σ(*F*^2^) is used only for calculating *R*-factors(gt) *etc*. and is not relevant to the choice of reflections for refinement. *R*-factors based on *F*^2^ are statistically about twice as large as those based on *F*, and *R*- factors based on ALL data will be even larger.

### Fractional atomic coordinates and isotropic or equivalent isotropic displacement parameters (Å^2^)

**Table:**

	*x*	*y*	*z*	*U*_iso_*/*U*_eq_	
Cl	0.02248 (4)	0.79411 (2)	0.19541 (2)	0.02921 (10)	
O1	0.18412 (16)	0.46030 (7)	0.46513 (7)	0.0383 (2)	
O2	0.27741 (17)	0.60583 (8)	0.49519 (6)	0.0414 (3)	
N	0.21538 (15)	0.54450 (7)	0.44328 (7)	0.0269 (2)	
C1	0.09994 (14)	0.68212 (7)	0.23325 (8)	0.0200 (2)	
C2	0.11332 (14)	0.66404 (7)	0.32692 (7)	0.0202 (2)	
C3	0.18229 (14)	0.57217 (8)	0.34792 (7)	0.0198 (2)	
C4	0.22965 (15)	0.50274 (8)	0.28388 (8)	0.0207 (2)	
H4A	0.2734	0.4412	0.3026	0.025*	
C5	0.21212 (15)	0.52466 (8)	0.19236 (7)	0.0218 (2)	
H5A	0.2439	0.4783	0.1474	0.026*	
C6	0.14754 (15)	0.61522 (8)	0.16681 (7)	0.0218 (2)	
H6A	0.1360	0.6313	0.1041	0.026*	
C7	0.05257 (18)	0.73682 (9)	0.39679 (9)	0.0294 (3)	
H7A	−0.0502	0.7744	0.3725	0.044*	
H7B	0.1544	0.7804	0.4111	0.044*	
H7C	0.0139	0.7032	0.4523	0.044*	

### Atomic displacement parameters (Å^2^)

**Table:**

	*U*^11^	*U*^22^	*U*^33^	*U*^12^	*U*^13^	*U*^23^
Cl	0.02669 (16)	0.01774 (15)	0.04320 (19)	−0.00006 (9)	−0.00430 (11)	0.00518 (11)
O1	0.0576 (6)	0.0269 (5)	0.0303 (5)	−0.0020 (4)	0.0027 (4)	0.0077 (4)
O2	0.0613 (7)	0.0399 (6)	0.0232 (4)	−0.0141 (5)	−0.0070 (4)	−0.0023 (4)
N	0.0321 (5)	0.0269 (5)	0.0218 (4)	−0.0029 (4)	0.0012 (4)	0.0015 (4)
C1	0.0176 (4)	0.0150 (4)	0.0274 (5)	−0.0011 (4)	−0.0013 (4)	0.0010 (4)
C2	0.0185 (5)	0.0174 (5)	0.0248 (5)	−0.0030 (4)	0.0024 (4)	−0.0032 (4)
C3	0.0208 (5)	0.0197 (5)	0.0189 (5)	−0.0033 (4)	0.0009 (4)	0.0007 (4)
C4	0.0207 (5)	0.0159 (4)	0.0256 (5)	−0.0006 (4)	0.0003 (4)	−0.0015 (4)
C5	0.0221 (5)	0.0202 (5)	0.0232 (5)	−0.0012 (4)	0.0013 (4)	−0.0054 (4)
C6	0.0222 (5)	0.0226 (5)	0.0207 (5)	−0.0027 (4)	−0.0013 (4)	−0.0003 (4)
C7	0.0326 (6)	0.0234 (5)	0.0324 (6)	−0.0011 (5)	0.0074 (5)	−0.0095 (5)

### Geometric parameters (Å, °)

**Table:**

Cl—C1	1.7410 (11)	C4—C5	1.3832 (16)
O1—N	1.2300 (13)	C4—H4A	0.9500
O2—N	1.2275 (14)	C5—C6	1.3907 (16)
N—C3	1.4713 (14)	C5—H5A	0.9500
C1—C6	1.3891 (15)	C6—H6A	0.9500
C1—C2	1.4010 (15)	C7—H7A	0.9800
C2—C3	1.4018 (15)	C7—H7B	0.9800
C2—C7	1.5045 (15)	C7—H7C	0.9800
C3—C4	1.3882 (15)		
			
O2—N—O1	124.17 (11)	C3—C4—H4A	120.6
O2—N—C3	118.13 (10)	C4—C5—C6	119.41 (10)
O1—N—C3	117.66 (10)	C4—C5—H5A	120.3
C6—C1—C2	123.55 (10)	C6—C5—H5A	120.3
C6—C1—Cl	116.78 (8)	C1—C6—C5	119.74 (10)
C2—C1—Cl	119.66 (8)	C1—C6—H6A	120.1
C1—C2—C3	113.76 (10)	C5—C6—H6A	120.1
C1—C2—C7	121.93 (10)	C2—C7—H7A	109.5
C3—C2—C7	124.29 (10)	C2—C7—H7B	109.5
C4—C3—C2	124.64 (10)	H7A—C7—H7B	109.5
C4—C3—N	115.04 (10)	C2—C7—H7C	109.5
C2—C3—N	120.29 (10)	H7A—C7—H7C	109.5
C5—C4—C3	118.87 (10)	H7B—C7—H7C	109.5
C5—C4—H4A	120.6		
			
C6—C1—C2—C3	0.94 (15)	O1—N—C3—C4	−38.35 (15)
Cl—C1—C2—C3	−178.30 (7)	O2—N—C3—C2	−38.54 (16)
C6—C1—C2—C7	−177.26 (11)	O1—N—C3—C2	143.61 (12)
Cl—C1—C2—C7	3.50 (15)	C2—C3—C4—C5	1.31 (17)
C1—C2—C3—C4	−1.68 (15)	N—C3—C4—C5	−176.63 (10)
C7—C2—C3—C4	176.46 (11)	C3—C4—C5—C6	−0.09 (17)
C1—C2—C3—N	176.17 (9)	C2—C1—C6—C5	0.13 (17)
C7—C2—C3—N	−5.69 (16)	Cl—C1—C6—C5	179.39 (8)
O2—N—C3—C4	139.50 (12)	C4—C5—C6—C1	−0.59 (17)
